# Spatiotemporal Distribution and Analysis of Organophosphate Flame Retardants in the Environmental Systems: A Review

**DOI:** 10.3390/molecules27020573

**Published:** 2022-01-17

**Authors:** Sinozuko Hope Bika, Abiodun Olagoke Adeniji, Anthony Ifeanyi Okoh, Omobola Oluranti Okoh

**Affiliations:** 1 Department of Chemistry, University of Fort Hare, Alice 5700, South Africa; ookoh@ufh.ac.za; 2 South African Medical Research Council (SAMRC) Microbial Water Quality Monitoring Centre, University of Fort Hare, Alice 5700, South Africa; aokoh@ufh.ac.za; 3 Department of Chemistry and Chemical Technology, Faculty of Science and Technology, National University of Lesotho, Lesotho P.O. Roma 180, South Africa; adenijigoke@gmail.com; 4 Department of Environmental Health Sciences, University of Sharjah, Sharjah P.O. Box 27272, United Arab Emirates

**Keywords:** organic pollutants, organophosphate flame retardants, aquatic environment, carcinogens, toxicity, endocrine system disruptions

## Abstract

In recent times, there has been a cumulative apprehension regarding organophosphate flame retardants (OPFRs) owing to their high manufacturing and usage after brominated flame retardants were strictly regulated and banned from being distributed and used in many countries. OPFRs are known as the main organic pollutants in the terrestrial and aquatic environment. They are very dangerous to humans, plants and animals. They are also carcinogenic and some have been implicated in neurodevelopmental and fertility challenges. OPFRs are distributed into the environment through a number of processes, including the usage, improper disposal and production of materials. The solid phase extraction (SPE) method is suggested for the extraction of OPFRs from water samples since it provides high quality recoveries ranging from 67% to 105% and relative standard deviations (RSDs) below 20%. In the same vein, microwave-assisted extraction (MAE) is highly advocated for the extraction of OPFRs from sediment/soil. Recoveries in the range of 78% to 105% and RSDs ranging from 3% to 8% have been reported. Hence, it is a faster method of extraction for solid samples and only demands a reduced amount of solvent, unlike other methods. The extract of OPFRs from various matrices is then followed by a clean-up of the extract using a silica gel packed column followed by the quantification of compounds by gas chromatography coupled with a mass spectrometer (GC–MS) or a flame ionization detector (GC-FID). In this paper, different analytical methods for the evaluation of OPFRs in different environmental samples are reviewed. The effects and toxicities of these contaminants on humans and other organisms are also discussed.

## 1. Introduction

Water (H_2_O) is regarded as the most profuse compound on earth and an important need in all parts of life, both in sufficient quality and quantity. Unfortunately, the quality of this important resource has become a global challenge due to pollution, mainly as a result of human activities and modern ways of life. Water is an ideal medium for distributing OPFRs [1], a set of chemicals identified in municipal wastewaters, surface waters, runoff of stormwater and urban precipitation, with concentrations extending from medium to high [1]. OPFRs are defined as emerging flame retardants, which are used to reduce the ignition of building and consumer materials [2]. OPFRs are used worldwide as lubricants, antifoaming agents, plasticizers, flame retardants and hydraulic fluids in consumer and building materials [3]; [3,4] reported that OPFRs can be detected in environmental and biotic matrices (i.e., water, soil, sediments, plants, humans and animals). OPFRs can enter the environmental matrices through informal e-waste handling facilities (i.e., heating, leaching of acids and burning of materials that contain OPFRs) [5].

OPFRs can also be detected in several bio-resource materials such as bio-solids and compost-like output, which are found to be beneficial in some instances and can be recycled in agriculture for soil improvement and used as alternative bedding materials for livestock [6]. The use of TPHP, TCIPP and TDCPP has increased drastically over the years, and these are examples of persistent OPFRs in the environment that have revealed toxicity and bioaccumulation that calls for investigation within industrial/municipal bio-resources to food chains in the future [6].

**Figure 1:**
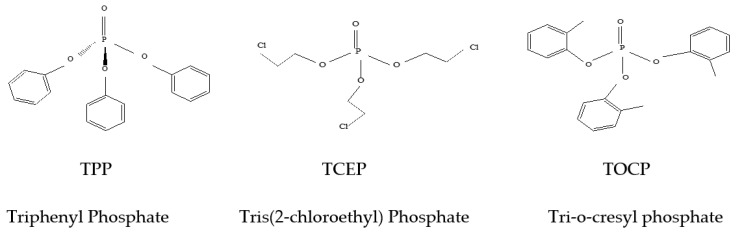
Typical examples of some major molecular structures of OPFRs [2].

Practices such as the production, extensive use, volatility and improper disposal of OPFRs are known causes for their appearance in environmental systems [7]. These pollutants are also regarded as semi-volatile organic compounds (SVOCs) that are mainly used in a variety of industries as plasticizers and flame retardants, and in the production of hydraulic fluids, foams, coatings of electronic appliances and defoamer agents to limit blazes due to their physicochemical characteristics [8]. Some of these OPFRs are shown in Figure 1.

OPFRs are often seen in parts of the environment and biota. These organic compounds are taking the place of brominated flame retardants and are raising new apprehensions considering their health and environmental risks. They are preferred as substitutes to polybrominated diphenyl ethers (PBDEs), which are well recognized toxicants [9]. PBDEs were considered to be persistent organic pollutants (POPs) [9,10] reported that the usage of OPFRs has steeply increased over the previous 15 years, suggesting high levels of human exposure to OPFRs rather than PBDEs.

OPFRs are commonly used and often found globally in several environmental matrices such as air, indoor dust, sediment, soil and water, as shown in Table 1. Frequently detected OPFRs include “Tris(2-butoxyethyl)phosphate (TBEP), Tris(2-Chloroethyl)phosphate (TRCP), Triphenyl phosphate (TPP), Tricresyl phosphate (TCP), Tributyl phosphate (TBP), Tris(2-ethylhexyl)phosphate (TEHP), Tris(2-chloroethyl)phosphate (TCEP), Tri-o-cresyl phosphate (TOCP), Tris(1,3-dichloro-2-propyl)phosphate (TDCPP) and Tris(1-chloro-2-propyl)phosphate (TCPP)” [11].

OPFRs are strongly bound covalently to materials that are regarded as hosts [12]; these compounds may perhaps be drawn-out into the surroundings quite easily through leaching, abrasion and volatilization because of their use and wide range of applications as additives [13]. Usage of these OPFRs in Europe numbered, according to statistical data up to 85,000 loads in the year 2005: 39,000 loads of non-halogenated forms and 46,000 loads of chlorinated forms. In 2007, the usage of chlorinated OPFRs had increased to 51,000 loads [8,14,15].

According to the study of [16], total organic carbon (TOC) that was discovered from soil samples was found to have a high occurrence of these pollutants. TOC is one of the good physicochemical parameters used to predict the distribution of OPFRs in soil and sediments [16]. When OPFRs are analysed from soil/sediment samples, the texture of soil/sediment varies from size to size (i.e., gravel, coarse grains, fine grains, coarse clay and fine clay). OPFRs are likely to bond with fine grains, whereas those with short chains are likely to be found in gravel depending on the physicochemical properties of each OPFR [16].

In this review paper, we set our sights on the discussion of the occurrence and distribution of OPFRs as a class. Furthermore, we reviewed OPFRs based on their fate in the environment, evidence of their hazardous health effects and levels of exposure amongst the common residents, and measured their levels indoors using some collected works of other authors.

## 2. Physicochemical Properties of OPFRs

The physiological/physicochemical properties of OPFRs differ slightly. OPFRs that can, in particular, dissolve in water include dimethyl methyl phosphonate (DMMP) and di-ammonium phosphate (DAP), whereas isodecyl diphenyl phosphate (IDPP) and trixylenyl phosphate (TXP) cannot form a homogeneous mixture when mixed in water [17]. When solubility falls, molecular weight rises. In some cases, where the hydrolysis half-life of compounds is equal, pollutants with lower molar mass are more likely to be discovered in aquatic environments than those with higher molecular weights, as determined by log Kow for each OPFR. Most OPFRs have positive log Kow values, indicating that they dissolve or combine more readily in lipids (lipophilic) than in water. The values of log Kow differ significantly between different groups of OPFRs. [18] The study provided a calculated value of log Kow of 9.8 for tetrakis (hydroxymethyl) phosphonium sulphate (THPS), compared to 10.6 for trioctyl phosphate. Physicochemical properties are significant factors that aid in determining the behaviour of OPFRs and evaluating their effects on organisms in a specific environment [8]. Volatile OPFRs with higher vapour pressures, such as TCEP, TEP and TBP, have a greater chance of escaping into thin air and settling on top of dust than heavier OPFRs. Higher molecular weight alkyl and aryl-OPFRs are hydrophobic (do not mix well in water), and they have similar bioaccumulation factors (BCFs) and affinity for soil/sediment samples [8].

Chlorinated OPFRs have been shown to be water soluble and are regarded as long-term threats to aquatic animals [7,8].

According to [19], there are essential physical and chemical properties of OPFRs that include numerous key parameters that tell us about the compound’s non-ionic properties, the behaviour of pollutants in the environment, and the organic chemical fate. As shown in Table 2, these properties include vapour pressure (Ps), solubility in water (SW), octanol–water (KOW), octanol–air (KOA), and air–water (KAW) constants connected to Henry’s law constant [20].

Degradation rates define environmental persistence and, as a result, are critical contributor factors to chemical outcome models.

Some authors used a writing strategy that included publishing experimental evaluations, report outcomes, studies and an online database to compile an archive for KOA and KAW. They discovered that two software programs are used as tools of estimating: SPARC On-Line Calculator 4.6 and EPISuite 4.1 [21,22]. These two programs provide more KOW and KAW values. EPISuite uses the HENRYWIN v3.20 component to calculate KAW values. The HENRYWIN estimations are judged incorrect as they are different from the P_S_/S_W_ estimates by being more significant than the factor of 10^5^. The P_S_/S_W_ ratio is also used in some cases by EPIWIN to evaluate K_AW_ value. From the published literature by [23], a third estimation tool is used to sum up the values of K_OW_. Degradation half-lives in air (*t*_1/2, air_), water (*t*_1/2, water_) and soil (*t*_1/2, soil_) are calculated completely by the AOPWIN v1.92a and BIOWIN v4.10 modules of the EPISuite platform [24].

## 3. Application of OPFRs

OPFRs are commercially manufactured to be used in different applications such as consumer products and building materials because of their broad spectrum of physiological and physical properties on earth. The bioaccumulation factor (BCF), vapour pressure (Ps), log K_ow_ value, solubility, solvent resistance, stable molecular properties and good water resistance are examples of these properties [2]. Most OPFRs are found in substances and by-products as additives and can reach high percentages of content. OPFRs are not bound to by-products of a substance chemically because they are easily released into the environment where they pose risks to humans, plants and animals [25].

Halogenated OPFRs are part of the flame retardants (FRs) family, though non-halogenated forms are regarded as plasticizers [8,26]. Phosphates such as TPHP, TiBP and TBP are regarded as non-derivatized alkyl phosphates and are applied as lubricants and plasticizers to synchronise porosity [26]. OPFRs such as TPP can be mixed with non-halogenated and halogenated flame-retardants from changed profitable combinations that are usually added to polyurethane foam. TPP has been mixed together with PentaBDE in foam. Some examples such as TBEP are used to make floor polish [18,26,27].

OPFRs can be used in the commercial production of foams, coatings, plastics, textiles and furniture because of their stable molecular properties [28]. China is described as the utmost consumer of OPFRs; studies have revealed that an amount of 78,000 tonnes was reached due to the high production and delivery capacity of OPFRs in 2013 [29]. OPFRs can be added to products instead of chemically bonded with them; hence, they are easily spread into the environment [30].

## 4. Sources of OPFRs

**Figure 2:**
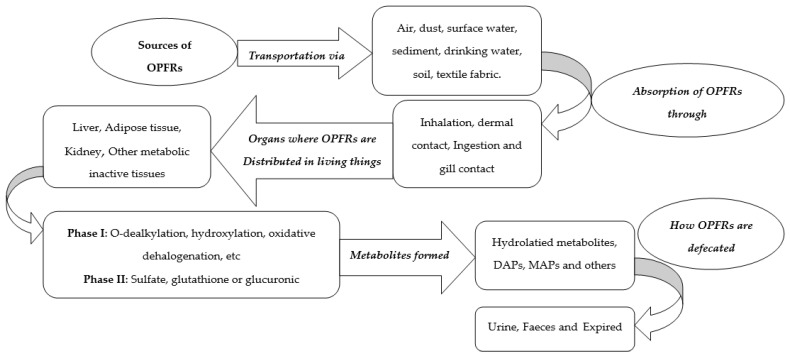
A diagram showing providence of OPFRs in animal and human bodies [33].

The process of distributing organic pollutants into the environment is recognised as a prominent issue with regards to their fate, environmentally. Wastewater treatment plants (WWTPs), sewage treatment plants (STPs), wastewater discharge from factories and atmospheric depositions from industries are marked trails for OPFRs being distributed into the terrestrial and aquatic environment [31,32]. H_2_O is a prime origin of OPFRs; hence, traces of these pollutants are seen in sewage, surface water, stormwater runoff and urban precipitation. The concentration range of OPFRs in water is from a medium to a high level. Figure 2 shows a mind map of how OPFRs are spread in humans and animals.

In previous years, OPFRs were found in distinctive deposition models from Sierra Nevada [34]. These phosphates were used to fight blazes in the woodlands of Sierra Nevada. TnBP and TCPP were found in interior air and dirt [12,35]. Computers were discovered to be major sources of indoor pollution by TPP [36].

## 5. Bioresources and Biocomposites of OPFRs

Cereal and associated crops have become reliable sources of phenols and alcohols used as precursors for the production of OPFRs that are friendly to the environment. They are renewable, readily available, cheap and not/less toxic. Examples of these include starch from many cereal crops, from where isosorbide, a diether diol, is obtained. Other examples include phenolic compounds from plant origin, e.g., 3,5-dihydroxybenzoic acid (from buckwheat) and 3,4,5-triydroxybenzoic acid (gallic acid, produced by numerous plants), which have great flame retardancy. These materials are less toxic compared to their organohalogen counterparts [30,37]. In fact, the toxicity levels of some simple phosphate esters are very negligible, except for them being combined with some halogens [38]. These FRs from bio-sources are known to resist fire by forming chars, which serve as a heat sink, thus, disallowing the transfer of heat to the bulk of the material. These bio-based FRs are sometimes added to polymers to boost their flame retardant properties. They also serve as a heat barrier and guard to composite surfaces against heat and air [39].

Biocomposites fortified with some bio-based materials such as vegetable fibres could be either partially friendly with the environment but non-biodegradable or completely environmentally friendly and also degradable biologically. These sets of biocomposites from plant fibre origin and biopolymers are thus referred to as green composites, given their environmental friendliness [40,41,42,43]. Examples of such include polyhydroxyalkanoates, starch, cellulose, chitin and chitosan, (polylactide), proteins, bio-polybutylene succinate, and poly (butylene adipate-co-terephtalate). Some of them have found applications in the production of degradable bags for shopping, coatings, biomedical and several plastic applications. Some composite materials are fortified with nanoparticles, which improve their mechanical, optical, thermal and/or electrical properties, thus making them degradable biologically and more friendly to the environment. Many of these materials are polymeric in nature and more commonly utilized in both the construction and automotive industries. Phosphorus-based additives have in recent times been preferred to the halogen additives [44,45,46,47]. These phosphorous containing materials are used to start the charring of polyol, in order to enhance its fire retardancy [48].

Commonly used additives with flame retarding properties include nitrogen-based compounds, mineral compounds, phosphorus-based compounds, halogenated compounds, nanometric particles and silicon-based compounds. Phosphorus-based materials such as red phosphorus, phosphate esters, phosphinates, phosphonates, and even inorganic phosphates such as ammonium polyphosphate (APP) first decompose to produce phosphoric acids, which condenses to produce phosphorylated structures and water. This in turn results in a carbonaceous layer of protection. These phosphorus-based materials can also turn into vapour or gas to produce some active radicals, which can scavenge both OH and H radicals. In some cases, these FRs can be combined with nanoclays to increase their fire retardancy. Phosphorus-based FRs are amongst the most patronized in the market now because they are seen as better alternatives to the halogen-based FRs in terms of their performance, toxicity and cost [49,50,51,52,53] (https://www.aimplas.net/blog/composites-fire-developments-new-trends-flame-retardant-additives/ accessed on 2 January 2022). Some organic and inorganic phosphorus compounds, such as triaryl phosphates, ammonium polyphosphate, phosphate esters, aluminium diethyl phosphinate, resorcinol bis(diphenyl phosphate) and melamine polyphosphate or melamine bis(diphenyl phosphate), have been found to be very effective as FRs [54]. However, they have to be used in large amounts when considered, although this would negatively affect their polymer properties in the process [55].

## 6. Toxic Effects of OPFRs and Risk Exposure

**Figure 3:**
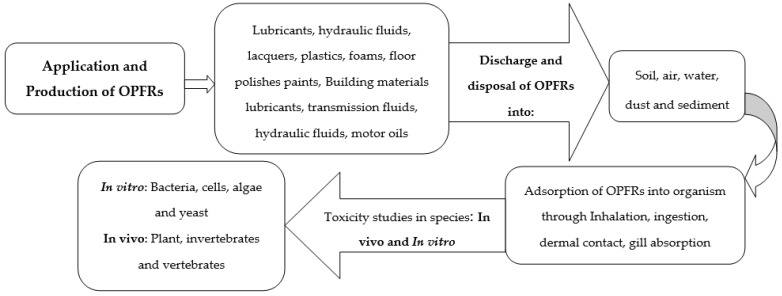
A schematic diagram of OPFRs action in toxicology [11].

OPFRs are widely used, are usually detected in different environmental matrices and have been found to be emerging contaminants [2,3]. The market trend of OPFRs has been continuously increasing and that has resulted in bioaccumulation and health harms [56]. Exposure to OPFRs can be by absorption, bioaccumulation and internal exposure [2]. These pollutants are used as additives through physical integration within the manufactured products; hence, they can be easily released into the environment through deposition, volatilization, dissolution, leaching, abrasion and infiltration [2,3]. According to [57], a class of 97 OPFRs was revealed as toxic using a model known as the “Quick Chemical Assessment Tool” (QCAT). Although certain flame retardants are considered important compounds by the US EPA, they need to be studied further to obtain more information or to predict their controlling measures. OPFRs in humans and animals have become everyday issues due to their behaviour, as shown in Figure 3.

### 6.1. Toxicity of OPFRs in Humans

OPFRs have been detected in humans, which has badly affected the health of humans in different ways [56]. According to the study of [58], humans in their everyday life are exposed to OPFRs through inhalation, dietary intake, ingestion, skin contact and dermal interaction in the dust and air that comprises traces of these pollutants. Ingesting dust particles and dermal/skin absorption were found to be the major ways of human exposure to these OPFRs [2]. OPFRs are found to be neurotoxic, carcinogenic and mutagenic in humans [59]. They also show systematic and endocrine disruptive effects [3,8,60]. The consumption of contaminated fishes can increase human exposure to these pollutants [3].

OPFRs are also found in children’s products, which pose great danger and has caused significant apprehension. The European Commission has approved values specifically for the existence of these pollutants in children’s toys, at a maximum of 5 mg kg^−1^ [58]. Therefore, it is necessary to have a better understanding of and protect humans when it comes to being exposed to these pollutants, and be familiar with their sources of emission [3]. The degree of sex hormones in people’s cells is usually disturbed by TDCPP [61,62].

### 6.2. Toxicity of OPFRs in Animals/Living Organisms

Due to the detection of OPFRs in organisms and their surroundings, cumulative attention is now being paid to their adverse effects. OPFRs in animals have been proven within the literature to affect and harm the reproduction, development and motor activity of *Planaria*, zebrafish, and *Caenorhabditis Elegans* [57]. Studies have proved that being exposed to OPFRs can cause neurodevelopmental toxicity in a range of in vitro tests, which indicate the dangerous effects to neurodevelopment (i.e., neurite outgrowth, synaptogenesis, proliferate of neurons and the system development in animals). Some compounds such as TnBP and TPP are neurotoxic, although some are carcinogenic to creatures, e.g., TDCP and TCEP in rats [8]. Additionally, in cultivated domestic fowl embryos, TDCPP initiated improved expression of TH-responsive genetic factors [14].

### 6.3. Risk Assessment of OPFRs

Risk assessments are conducted in studies relating to OPFRs in the environment by determining the hazard quotients (HQs), or risk quotients (RQs), as the case may be. This can be determined for water, soil, sediment or any aquatic organism such as fish. It is performed by taking the ratio of the measured environmental concentrations (MEC) of the FR to the predicted no effect concentration (PNEC) obtained from the literature, as represented in Equation (1)

RQ = MEC/PNEC(1)

PNEC represents the FR’s concentration thought to have no damaging effect on the DNA in the liver. When PNEC is derived from the chronic toxicity value, then a factor of 100 is used to divide. However, 1000 is the most commonly used factor when acute toxicity value is used [63]. PNEC is often determined using Equation (2) [31,64,65].

PNEC = L(E)C_50_/f (2)

where L(E)C50 represents LC50, which is the lethal concentration necessary to destroy or kill 50%, or EC_50_, which is the amount of a toxicant that will induce a response halfway between the baseline and maximum after a stated time of exposure, and f is the security factor. This assessment result could vary from low risk to high risk to the aquatic organisms in such an environment. Low risk is implied by 0.01 < RQ < 0.1; moderate risk by 0.1 < RQ < 1 and high risk by RQ > 1 [56,65,66,67,68,69]. Table 3 shows some PNECs for OPFRs in different environmental matrices.

## 7. OPFRs Analysis

Many researchers have reported the occurrence of OPFRs in numerous environmental media. The processes for clean-up, extraction, sample collection and OPFRs analytical techniques are similar to those used for analysing many other flame retardants. The sample collection, storage and extraction methods for some OPFRs in environmental media are presented in Table 4.

## 8. Extraction Methods for OPFRs in Different Environmental Media

Table 5 shows methods used for the extraction of certain OPFRs from certain matrices of the environment.

## 9. Analytical Procedures for OPFRs in Water and Sediments

Pantelaki and Voutsa [9] suggested a need to develop dependable logical methods that will permit a selective, sensitive and rapid determination of newly coming pollutants in samples from different parts of the environment [86]. Because of the flexible nature of substituents, OPFRs have varied properties, i.e., chemical and physical properties, starting from polar to more hydrophobic. The development of the method focuses on altering entire features to improve understanding and obtain precise quantification. Many methods for instrumental analysis are now obtainable in collected works for the determination of OPFRs in accessible and open water bodies, air, sediments and soil. OPFRs documented in previous studies include the tri-esters of OPFRs, chlorinated alkyl phosphates, alkyl phosphates and aryl phosphates. These contaminants were determined using GC or LC coupled with MS or selective detectors [9].

### 9.1. Gas Chromatographic Methods

GC methods are used to determine compounds with no polarity, using non-polar inert phases, commonly (5% phenyl)-methylpolysiloxane such as HP5 and DB-5MS. The column length ranges from 15 to 30 m; commonly used columns are 30 m length × 0.25 mm internal diameter × 0.25 μm film thickness [31,87]. There are complications when separating and detecting some OPFRs’ constituents depending on the physical properties of the inert phase. [88] confirmed the effectiveness of two duct columns with identical dimensions (30 m × 0.25 mm i.d., 0.25 μm film thickness) in comparison to the coated phases such as DB-5 ((5%-phenyl)-methylpolysiloxane) and SPB-1701 ((14%-cyanopropyl-phenyl)-methylpolysiloxane). The study reported quality results for most OPFRs, but for the depressed separation of TCEP and an isomer of TCPP in the SPB-1701 column (Supelco, Bellefonte, Pennsylvania, USA) and the temperature-dependent selectivity of TBOEP and TPHP in the DB-5 column. Finally, a DB-5 column is suggested to be the most appropriate column for wastewater and sediment study [9].

### 9.2. Liquid Chromatographic Methods

LC/MS is appropriate for determining OPFRs such as EHDPP, TEHP, TPHP, TnBP and TMPP, which are not vaporizing enough for analysis in GC [89]. LC or UPLC are passed out in a reversed-phase mode, mostly using C18 or C8 stationary phases (UPLC BEH C18, Luna C8, Waters xterra C18) or combined reversed-phase with hydrophilic interactions column (HILIC-1). Usually, in grade washes are used as mobile phases to separate reversed-phase OPFRs, water mixtures (acidified with formic acid, acetonitrile or methanol). LC-MS/MS or UHPLC-MS/MS methods using triple quadrupole mass analysers are often used to determine OPFRs in environmentally friendly samples because of their extraordinary selectivity and sensitivity. The examination is accepted using electrospray ionization (ESI) in negative or positive mode of ionization depending on multiple reaction monitoring (MRM) gaining modes [89].

### 9.3. Nitrogen Phosphorus Detector (NPD)

NPD is another analytical procedure used for determining OPFRs in water samples [87,88]. Flame photometric detector (FPD) is considered a substitute to NPD for its selectivity and sensitivity, which are quite similar to the former [90]. The use of mass spectrometry has its advantages in identifying these pollutants. Therefore, GC–MS or GC–MS/MS is used as a method lower than electron impact ionization in the selected ion monitoring (SIM) mode. Based on the study of [91], it is believed that NPD and FPD procedures present the same detectability for many OPFRs, although other literature preferred GC–MS/MS due to its better selectivity and detection limits [92,93]. Using chemical ionization, GC–MS is not adequate for many OPFRs except for TCPP that displays higher detectability [91,92]. The study of [94] recommended gas chromatography linked with a heated interface with inductively coupled plasma mass spectrometry (GC-ICP-MS) to determine OPFR samples. The NPD method delivers quality sensitivity and selectivity for the determination of P-containing molecules in environmental surroundings because of the combination of a pulsed splitless injection mode with low radio frequency power and solid extraction conditions [9].

## 10. Levels of OPFRs in the Environment across the Globe

Organophosphate flame retardants (OPFRs) are the third vital set of flame retardants besides brominated flame retardants and aluminium trihydrate, constituting about 14% of the global market [5,7]. With the large-scale and economic availability of many newly made reference standards and the advent of sophisticated analytical methods, all OPFRs are analysed from numerous environmental matrixes worldwide, including North America, Europe, Asia, Africa and China, to mention a few. These pollutants were detected in indoor air, outdoor air, sediment, soil, water, adipose tissues, blood and other human fluid samples, and in animals. OPFRs were detected in different environments around the world, indicating that these pollutants are ubiquitous, The global output of OPFRs increased from 186,000 tons in 2001 to 620,000 tons in 2013 [95]. Several adverse effects of OPFRs on the ecosystem and human health have been reported [95,96].

Table 6 summarises the levels of OPFRs that have been reported from different studies and in different matrixes around the world. The surge in demand for devices containing OPFRs accounts for their high concentrations in river water [97], due to indiscriminate dumping of products with a high level of contaminants, discharge of sewage, and municipal or industrial wastes not treated or partially treated [26]. In their study, [98] reported the highest OPFRs concentrations in sediments around the world. High OPFRs concentrations are found in creeks to offshore or offshore lake waters in that order because creek sediments act as a sink for these contaminants.

Higher concentrations of TDBPP are commonly reported because this compound has been used as a flame retardant in plastics, synthetic textiles and fibers, which have been included in children’s clothing [113]. Similarly, a higher concentration of TBP in the environment may be linked with antifoaming agent in concrete, as well as a wetting agent in casein glue and as a pasting agent in pigment paste, apart from using it as the primary ingredient in hydraulic fluids [114]. In China, the concentrations of OPFRs in different matrixes from urban to industrial areas are higher than those collected from rural and agricultural areas [30]. This might be because of high industrial development, great population density and huge amounts of OPFRs vaporised into thin air and wastewater from industrial areas. The region is surrounded by industries that manufacture items that contain traces of OPFRs. Many studies have been conducted in China to greatly address the organic phosphate pollution problem worldwide. The fact remains, if the use of plastics increases and industries that produce materials containing OPFRs are not properly monitored, then the world should prepare for the consequences on the lives of humans, plants and animals.

Research conducted by [5] established a system that can analyse muscles of fish samples with traces of OPFRs. In the study, OPFRs found in animals were below the method detection limit. These low concentrations may be connected with various ways of living and diverse feeding habits [5]. It is known that physicochemical parameters of sediments, such as total organic carbon (TOC), can impact the levels of OPFRs [73].The difference in contamination profiles with sample matrixes is due to the hydrophobicity of a particular OPFR [98].

## 11. Conclusions

Based on previous studies, the environment has been proven to be contaminated with OPFRs due to their continuous production and the use of compounds with traces of OPFRs, improper disposal, indoor/outdoor dust, waste discharge from municipal water, waste from industries, dispersal of suburban and urban stormwater and sewage outfalls. The exposure of humans, plants and animals to OPFRs in everyday life has shown a significant impact on the aquatic and terrestrial environment. Even though there is no guideline yet for these deadly pollutants, researchers have shown that the way in which people and other creatures consume these pollutants is hazardous and can cause long and short term health effects on the body. Moreover, they are known to be possible endocrine disrupting compounds. The SPE method was established to be the best method used for the extraction of these pollutants from water samples and for aqueous clean-up because of its good recoveries and acceptable relative standard deviations. In the same vein, MAE has been proven to be a more effective method for extracting OPFRs from solid samples, considering its good recoveries and RSDs, being a faster method and requiring less solvent than other methods. The concentrations of OPFRs were greater than those of BFRs when analysing samples from schools, offices and family residences. OPFRs are mostly found in polyurethane (PU), construction, foam, construction refrigerators, electronics and electrical equipment. Therefore, it is noteworthy that higher levels of contaminants are recorded when tides are low, possibly due to a large inflow of effluents, urban runoff, stormwater and industrial discharge into an aquatic body. Aquatic environments next to mines and industries appear to be areas where high levels of these pollutants are usually found, hence, they are not suitable for people to live.

## Figures and Tables

**Table 1:** A compilation of some major OPFRs with MF, MW (g/mol) [5,7,11].

OPFRs	Full Name	MF	MW (g/mol)
TPP	Triphenyl phosphate	C_15_H_33_O_4_P	308.4
TBP	Tributyl phosphate	C_12_H_27_O_4_P	266.3
TBOEP	Tris (2-butoxyethyl) phosphate	C_18_H_39_O_7_P	398.5
TRCP	Tris (2-chloroethyl) phosphate	C_6_H_12_Cl_13_O_4_P	285.5
TEHP	Tris(2-ethylhexyl)-phosphate	C_24_H_51_O_4_P	435.0
TCPP	Tris(1-chloro-2-propyl)-phosphate	C_9_H_18_Cl_3_O_4_P	327.6
TOCP	Tri-o-cresyl phosphate	C_21_H_21_O_4_P	368.4

Keywords: MF = Molecular Formula, MW = Molecular Weight.

**Table 2:** Table showing the list of some major OPFRs with their water solubility, adsorption to soil and sediment, bioaccumulation factor and octanol–water coefficient [9,11,14].

OPFRs	Henry’s Law Constant (atm.m^3^/mol)	Molecular Weight (g/mol)	Water Solubility (mg/L) at 25 °C;	Vapour Pressure (mm/ Hg)	Log K_OW_	Bioaccumulation Factor (BCF)
TRCP	1.67 × 10^−7^	285.5	7000	0.061	1.63	0.425
TBP	1.4 × 10^-6^	266.32	280	1.13 × 10^−3^	4.00	39.81
TBOEP	1.2 × 10^−11^	398.5	1.100	0.03	3.00	25.56
TEHP	2.38 × 10^−2^	434.6	0.6	8.25 × 10^−8^	9.94	3.162
TCEP	1.67 × 10^−7^	250.2	7000	0.061	1.63	0.425
TOCP	9.21 × 10^−7^	368.4	0.3	1.10 × 10^−7^	6.34	2534
TDCPP	2.61 × 10^−9^	430.9	7.0	2.61 × 10^−9^	3.65	21.4
TCIPP	4.69 × 10^−7^	327.6	1200	5.64 × 10^−5^	2.89	3.27

**Table 3:** Some OPFR congeners with PNEC (ng/g) from China in different matrices.

Congener	Matrices	PNEC (ng/g)	References
TCEP	Carassius auratus auratus Soil	90,000 386	[65]
TCPP	Carassius auratus auratus Soil	30,000 1700	[65]
TCIPP	Carassius auratus auratus Soil	5100 320	[65]
TMP	Pimephales promelas Soil	7000	[65]
TCrP	Carassius auratus auratus Soil	110 -	[65]
TnBP	Carassius auratus auratus Soil	880 -	[65]
TiBP	Carassius auratus auratus Soil	20 -	[65]
EHDPP	crustacean Soil	18 302	[65]
TPHP	Carassius auratus auratus Soil	700 130,000	[65]

**Table 4:** Table showing the list of some matrices and how they are sampled, stored and extracted.

Type of Matrix	Example of Sites	Sample Collection	Storage	Extraction Method	References
Air	Private homes, indoor microenvironments, offices, day-care centres, private cars, schools, building material markets and floor/carpet stores	Vacuum pump connected with a gas meter	Quartz Fibre Filter (QFF) and Polyurethane Foam Plug (PUF- PAS) covered with aluminium foil.	Ultrasonic bath	[58]
Water	Waste water treatment plants (WWTPs), rivers, taps, surface water, sea and dams	Pre-cleaned 1 Litre amber glass bottle	Ice chest at 4 °C	Solid Phase Extraction	[3]
Sediments Soil	Dumpsite, river and terrestrial	Grab sampler Metallic spoon	Sealed in aluminium foil and stored in an ice chest	Ultrasonic bath, Ultrasound Assisted Extraction (UAE), Liquid–Liquid Extraction (LLE)and Microwave-Assisted Extraction (MAE)	[70] [71] [3]
Fishes/Other biota	Water environment	Gill or trap netting, electrofishing, tangling, gilling, filtering, spearing and pumping	Samples are preserved on dry ice	Soxhlet extraction (SE), Pressurised Liquid Extraction (PLE)	[72] [73]
Urine Breast milk Blood	Human	Metallic container Passive breast milk sampler or breast pump Syringe, needle and vein puncture	Pre-cleaned glass bottles	Solvent-induced phase transition extraction (SIPTE) Solid Phase Extraction (SPE)	[74] [56]

**Table 5:** Extraction methods used for OPFRs from different matrices, their advantages and disadvantages.

Extraction Method	Advantages	Disadvantages	Matrices that Can Be Extracted	References
Liquid–liquid extraction (LLE)	Remove inorganic compounds and can be used to deprotonate or protonate acids and bases	Challenging, time-wasting and demanding multiple extractions	Blood Water	[75] [76]
Ultrasonic assisted extraction (UAE)	Low-cost, appropriate, and suitable substitution to other extraction methods	Variables associated with UAE (i.e., frequency, power time etc) needs to be optimized for each product	Sediments Marine algae Fruit and Vegetables	[77] [78] [79] [80]
Microwave-assisted extraction (MAE)	Decrease the amount of solvent used and time, enhances reproducible results and helps in retrieving analytes from samples	To obtain results for OPFRs combine it with gel permeation chromatography and silica gel	Lipid samples	[2] [5]
Soxhlet extraction (SE)	Affordability and ease of operation, uninterrupted distinct method	Consumption of large volume of solvent, time-consuming and labour intensive	Solid samples (Sediments and soil)	[81] [82] [83]
Solid phase extraction (SPE)	Low consumption of solvent, efficient, cheap, convenient operation and short time-consuming.	Poor selectivity	Water, milk	[84]
Accelerated solvent extraction (ASE)	Uses less solvent, less extraction time, high throughput and automatic operation	It is costly	Solid samples, biotic matrices and food samples	[85]

**Table 6:** Reported OPFR levels around the world from different matrixes using different extraction techniques and different analytical instruments.

Location	Sample Matrix	Congener	Concentration	Extraction Method	Instrument	Reference
Spain	Wastewater Sludge	10 OPFRs congeners	3.67–50 µgL^−1^ 35.3–9980 ng g^−1^dw	a b	A	[97]
China	Rice	6 OPFRs congeners	0.004–287 ng/g	c	B	[30]
Qinzhou Bay	Sea water Sediments	11 OPFRs congeners	150–885 ng/L 32.3 ng/g dw	a d	C	[3]
Beijing of China	Wastewater Sludge	10 OPFRs congeners	600–838 ng/L	a f	K	[99]
Shanghai	Urine	3 OPFRs congeners	0.05–2.10 ng/mL	a	K	[56]
South Africa (Vaal River)	Sediment	12 OPFRs congeners	68–278 ng g^−1^ dw	d	C	[71]
China	Soil Outdoor dust	12 OPFRs congeners	37.7–2100 ng/g 9.14–42.700 ng/g	d	C	[100]
Sweden	Indoor air	TCEP	310 ± 560 pg m^−3^	e	C	[101]
China (Controlled environment growth)	Wheat (*Triticum aestivum* L.)	14 OPFRs congeners	0.18–0.37 μg/g	f	C	[102]
Korean coast	Sediment Bivalves	18 OPFRs congeners	2.18–347 ng/g dw 6.12–206 ng/g dw	f	B	[103]
China	Seawater	4 OPFRs congeners	91.87–1392 ng/L	a	D	[104]
Europe (European River basin)	Sediment Fish	14 OPFRs congeners	0.25–34.0 ng/g dw 9.32–461 ng/g lw	g	B	[73]
Nepal	Soil	8 OPFRs congeners	25–27,900 ng/g dw	e	C	[105]
Northern China (Beijing)	Farmland soil	12 OPFRs congeners	0.543 μg/kg–54.9 μg/kg	d	E	[106]
South China	e-waste (Thermal treatment) e-waste (Open burning)	11 OPFRs congeners	3.70 × 10^4^–3.65 × 10^5^ ng g^−1^ 5.22 × 10^3^–9.27 × 10^4^ ng g^−1^	d	C	[107]
Canada (Ontario)	Surface water Wastewater	12 OPFRs congeners	1.5–30 ng/L	_	F	[108]
Austria	Wastewater Surface water Sediments	9 OPFRs congeners	4.1 and 13 ng/L 2.6 and 7.9 ng/L 0.48 and 11 μg/kg	h d	E G F	[109]
Korea	Drinking water	TCEP TCPP TBEP	<MDL-1660 ng/L	h	H	[110]
Korea (Shihwa lake)	Water Sediment	18 OPFRs congeners	28.3–16,000 ng/L 2.99–3800 ng/g dw	h e	B	[98]
South Korea (Nakdong River)	Fish (Crusian carp)	9 OPFRs congeners	Liver: 6.2–18.1 ng/g ww Muscle: 4.2–7.8 ng/g ww	d	C	[111]
China (Chengdu)	Surface water Sediment Wild fish Groundwater	13 OPFRs Congeners	19.1–533 ng L^−1^ 12.50–253 ng g^−1^ 114–2108 ng g^−1^ lw 11.7–149 ng L^−1^	a d e	I	[81]
Spain	Water Sediment	10 OPFRs congeners	0.0076–7.2 μg L^−1^ 3.8–824 μg kg^−1^	a e	I J	[31]
China	Rare minnows (*Gobiocypris rarus*)	TPHP TBOEP TDCIPP	0.012 and 0.12 mg/L 0.24 and 2.4 mg/L 0.04 and 0.4 mg/L	i	D	[112]

a-SPE (Solid Phase Extraction); b-SLPE (Solid–Liquid Phase Extraction); c-MAE (Microwave-Assisted Extraction); A-Advanced Oxidation Processes (UV/H2O2 and O3); B-GC–MS/MS (Gas chromatograph–triple quadrupole mass spectrometer); d-UAE (Ultrasound-Assisted Extraction); C-GC–MS (Gas chromatograph–mass spectrometer); e-SE (Soxhlet Extraction); f-ASE (Accelerated Solvent Extraction); D-UPLC-MS/MS; g-PLE (Pressurised Liquid Extraction); E-LC-MS (Liquid Chromatography–Mass spectrometer); F-LC-MS/MS (Liquid Chromatography–tandem Mass Spectrometry); G-HPLC; h-LLE (Liquid–Liquid Extraction); TCEP-tris(2-chloro ) phosphate; TCPP-tris(2-chloroethyl) phosphate; TBEP-tris(2-butoxyethyl) phosphate; H-GC/MSD (Gas Chromatograph–Mass Spectrometric Detector); I-GC–EI-MS/MS; J-GC–EI(ECNI)-MS; TPHP-triphenyl phosphate; TBOEP-tris-(2-butoxyethyl) phosphate; TDCIPP-tris-(1,3-dichloro-2-propyl) phosphate; i-Gravimetric technique; K- HPLC-MS (high performance liquid chromatography–mass spectrometry).
